# Diabetic and cardiovascular patients’ willingness to pay for upcoming national health insurance scheme in Côte d’Ivoire

**DOI:** 10.1186/s13561-019-0225-y

**Published:** 2019-03-08

**Authors:** Agbaya Stéphane Serge Oga, Akissi Régine Attia-konan, Fulgence Vehi, Jérôme Kouame, Kouamé Koffi

**Affiliations:** 1 Département de Santé Publique, Hydrologie et Toxicologie, UFR Sciences Pharmaceutiques et Biologiques, Université Félix Houphouët-Boigny, Abidjan, Côte d’Ivoire; 2 Service Information Médicale, Institut de Cardiologie d’Abidjan, Abidjan, Côte d’Ivoire; 3 Centre de Recherche et d’Etudes en Populations Politiques et Systèmes de Santé, Institut National de Santé Publique, Abidjan, Côte d’Ivoire; 4 Service de Pharmacie, CHU Cocody, Abidjan, Côte d’Ivoire

**Keywords:** Willingness to pay, Health insurance scheme, Diabetes, Cardiovascular diseases, Côte d’Ivoire, West Africa

## Abstract

**Background:**

Côte d’Ivoire’s current health care financing system results from successive reforms undertaken with government funding and international support. The country is moving towards a national compulsory health insurance scheme. This context offered an opportunity to study additional features of health insurance’s potential market in Sub-Sahara Africa developing economy. This study examined patients’ willingness to pay in order to get access to health care when it is needed.

**Methods:**

A cross-sectional study was carried out in four tertiary level teaching hospitals from October to December 2017. These hospitals are located in Bouake (service of cardiology) and in Abidjan (two services of Endocrinology-Diabetology and Institute of Cardiology). Monthly willingness to pay was elicited using the contingent valuation method through a bidding game pre-tested interviewer-administered questionnaire. Multinomial logistic regression analysis was performed to predict participants’ willingness to pay.

**Results:**

Out of 450 participants included in the analysis, 22.2% were not willing to pay at least 4.5 euros per month while 7.6%, 26.9%, 29.6%, 5.3% and 8.4% stated to be willing to pay 4.5, 7.5, 15, 30, and 45 euros per month, respectively. Males were 2.3 and 2.5 times more likely to be willing to pay 4.5 or 7.5 and 30 or 45 euros, respectively. However, there was no statistically significant difference between males and females who stated being willing to pay a premium of 15 euros per month as compared to the participants in the reference modality, below 4.5 euros.

**Conclusions:**

The findings indicated that the amount that participants were willing to pay is consistent with other previously elicited. The association of sex with the willingness to pay suggested what might influence the acceptability of and the contribution to the upcoming compulsory health insurance scheme. These pointed out that some market features have to be understood for a successful implementation of this social health insurance scheme.

**Electronic supplementary material:**

The online version of this article (10.1186/s13561-019-0225-y) contains supplementary material, which is available to authorized users.

## Background

WHO (World Health Organization) Africa Region stresses politics and health professionals to tackle increasing global burden and threat of non-communicable diseases, such as diabetes and cardiovascular diseases, on morbidity, disability, and mortality among sub-Saharan Africa people [1]. WHO outlined to achieve at least 50% of eligible people receiving drug therapy and counselling (including glycaemia control) to prevent heart attacks and strokes as well as 80% availability of the affordable basic technologies and essential medicines, including generics, needed to treat major non-communicable diseases in both public and private facilities [2]. Low use of key medicines is related to their unavailability and poor affordability for a large proportion of communities and households in upper-middle-income, lower-middle-income, and low-income countries [3]. The 2005 and 2011 World Health Assemblies urged the states members to ensure that health-financing systems include a method for prepayment of financial contributions for health care, with a view of risk sharing among the population to avoid catastrophic health care expenditure and individuals’ impoverishment as a result of seeking care. Even if Universal Coverage is a legitimate aspiration and a working concept consistent with the goal of health for all and its strategy of primary health care, many challenges exist in this quest, especially implementing pre-payment arrangements [4].

Côte d’Ivoire adopted Bamako Initiative in the 1990s as a result of health sector restructuration to face difficulties encountered by the country in the 1980s. This led to user fees and other out-of-pocket expenditures on health. The MUGEF-CI (Mutuelle générale des fonctionnaires et agents de l’État de Côte d’Ivoire) has the longgest social health insurance experience. It was created in 1973 in the public sector after the termination of free health care for civil servants. The MUGEF-CI has been managed by civil servants’ unions and associations since 1989. It counts 750,042 beneficiaries representing 3.3% of the whole resident population. The amount of contribution is 3% of the basic salary, pension or lifetime allowance up to a maximum of 7004 FCFA (Franc Communauté Financière d’ Afrique) (10.5 euros) per member per month to cover pharmaceuticals and dentistry. Employment-based health Insurance is being undertaken in diverse ways to cover private-sector employees. Côte d’Ivoire has been dealing with a law for a mandatory health insurance scheme since 2014 in order to comply with WHO’s recommendations and Ouagadougou 2008 Declaration on Primary Health Care and Health Systems in Africa. The pilot phase of implementation of this health insurance scheme started in July 2017 with students from public universities. Since resource mobilization was set on a contribution of 1000 FCFA (1.5 euros) per person per month, expenditure planning is based on actuarial calculations and selection of healthcare services and goods to be provided. Yet, little is known about public preferences and support for healthcare financing system in the country. So a study on the willingness to pay a government’s health insurance plan using the contingent valuation method could help set insurance premiums to provide substantial income for health goods and services with a more efficient and socially equitable health system [5, 6].

This study was carried out to elicit the willingness to pay for the upcoming national health insurance scheme among patients admitted to the diabetology or cardiology services of the university hospitals in Côte d’Ivoire (West Africa).

## Methods

### Study setting and patients selection

Côte d’Ivoire is a West African country ranked in 2016 according to the World Bank in the lower-middle-income countries with a low Human Development Index (0.474 under the fixed cutoff point 0.550) and a Gini coefficient of 43.2 (0 represents absolute equality - 100 absolute inequality). Its population was estimated to 22,671,331 inhabitants according to 2014 census. Less than 10% of the above population was covered by health insurance. In 2015, the proportion of people living below the poverty line (410.20 euros or 269,075 FCFA / year) was estimated to 46.3%. The distribution of the population was 49.7% in rural areas; 30.9% in urban areas and 19.4% in Abidjan [7, 8]. The health system is organized in two-sided pyramidal form, one administrative and one other service provider. This service provider side is divided into three levels in the public sector: 1910 first contact health centers and 68 district reference hospitals at the first level, 17 regional hospitals and 2 specialized hospital centers at the second level and 4 university teaching hospitals and 5 national specialized institutes at the third level [9]. This health system was put in enormous strain by the troubles which occurred in the country from 2002 to 2011. The total health expenditure in 2015, representing 5.86% of GDP (Gross Domestic Product), was estimated to 74.81 euros or 49,073 FCFA per capita, and households contributed 32.55% (National Health Accounts, 2015).

The study was carried out in four facilities: one regional and university teaching hospital in Bouaké, in the Centre of the country, where study took place in the service of Cardiology, and into Abidjan, the economic capital in the south of the country, in the service of Endocrinology-Diabetology of two university teaching hospitals and in the Institute of Cardiology.

In the month of October 2017, patients attending these four public facilities for diabetes or cardiovascular diseases were requested to complete an anonymous demographic form (Additional file 1) after obtaining their verbal consent. Included patients were aged 18 and above, diagnosed with the target diseases at least 6 months before the survey. Patients with diabetes or hypertension linked to pregnancy were excluded. The survey focused on the adult patients even if they said that their health expenditure was paid by another person because everyone in the age of adulthood is expected to enroll for the upcoming national health insurance scheme in Côte d’Ivoire. Thus, it was worthwhile to take into account the position of each one. This study was approved by the Ethics Committee of “Institut de Cardiologie d’Abidjan”.

### Data collection

**Fig. 1:**
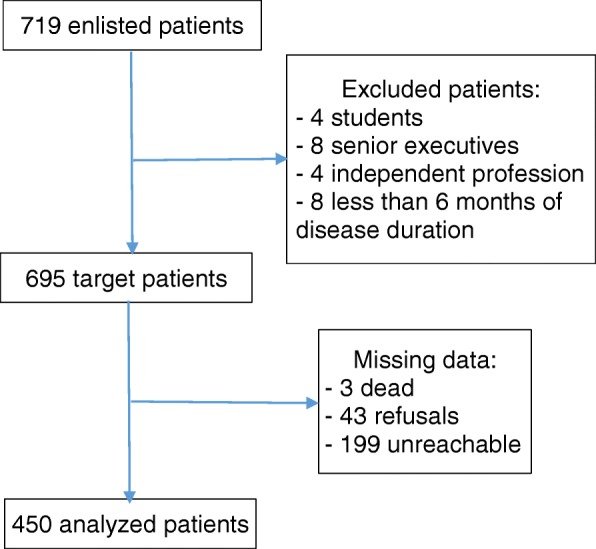
Chart flow of patients selection for analysis

Data were collected in French by two trained ladies, master graduate students from October to December 2017. A pre-tested interviewer-administered questionnaire (Additional file 2) was used to collect data by phone from the participants. For illiterate participants, a relative was requested for translation in conducting the phone interview. The two ladies involved in the data collection attended a three days comprehensive training workshop in how to conduct the questionnaire. The aims of the study were explained to them and all the questions in the questionnaire instrument were highlighted. They read, managed the questionnaire, and recorded the participants’ responses using a computerized anonymous form. A review of the recorded data was implemented every week in order to check completeness and consistency of the records. Whenever needed, participants were called again to check the accuracy of recorded responses. All the missed participants were called at least twice to minimize non-participation before considering them unreachable. Some respondents refused to begin or to continue the interview. The main reason given for refusing to take part in the study was that they did not trust the project of a national health insurance scheme. Other participants explained their inability to pay by the lack of stable income or the increase of monthly domestic expenditure. Sixteen participants corresponding to three social categories (students, senior executives, and independent profession) were excluded due to a low number. The process leading to analyzed patients is drawn in Fig. 1.

### Eliciting willingness to pay

The study questionnaire was designed to elicit the participant’s willingness to pay using the contingent valuation method. Participants were presented with the following hypothetical scenario: “Supposing that the public health insurance covers the costs of consultations, hospitalizations, laboratory tests, radiography, medicines, and surgical operations all depending on your subscription”. Then, respondents were asked whether they would be willing to pay a monthly health insurance premium in order to benefit from ensuring the sustainability of the healthcare services listed above. The starting point of the bidding game was set at 15 euros. The following amounts were 7.5 then 4.5 or 45 then 30 euros according to the respondent’s rejection or approval of the previous amount. These amounts set for WTP were based on premiums currently paid per month for public or private health insurance schemes in the country. The public scheme Ivoir’ Santé claims 22.5–37.5 euros added to MUGEF-CI basic plan (up to 10.5 euros) per family of maximum 10 people. In the private schemes, premiums vary and may exceed 150 euros per family of 5 people. The respondents who rejected 4.5 euros were considered not willing to pay any substantial premium.

### Data analysis

The patients’ characteristics were described, and the participants and non-participants were compared using sex, age and disease duration as criteria. The chi-square test was used. The distribution of the participants according to the amount of premium they were willing to pay was drawn then the association of this amount of premium with the participants’ characteristics was tested using a chi-square test. Multinomial logistic regression analysis was performed to predict the participants’ willingness to pay. This econometric analysis was conducted based on the following equation of modeling:

$$ \mathit{\ln}\frac{P\left(Y=k/X\right)}{P\left(Y=K/X\right)}={a}_{0,k}+{a}_{1,k}{X}_1+\dots +{a}_{j,k}{X}_j $$

*k = 2,…,K.; k = 1 is the reference. K is the number of modalities of the response variable (Y); j is the number of independent variables (X).*

The dependent variable, amount of premium, was recoded into four modalities, below 4.5, 4.5 or 7.5, 15 and 30 or 45 euros and the first one was taken as reference. For each independent variable, the last category was taken as reference. Modeling was carried out using a backward stepwise procedure based on Wald statistics with entry and removal limits fixed at 0.05 and 0.1, respectively. The model fit was assessed by computing the log-likelihood then the coefficients were estimated and odds ratios with 95% confidence intervals were derived. All analyses were carried out using SPSS 18.0 software and statistical tests were two-sided with a significant level of 0.05.

## Results

Out of the 719 patients enlisted, 3 had died before the interview, 43 (6%) refused to participate and 199 (28%) could not be reached despite three attempts. The patients’ demographic and duration of illness data are shown in Table 1. The 206 patients in the 5–47 years of duration were aged from 21 to 88 years with a median of 60. The mean (standard deviation) and median (IQR) durations were 12.4 (8.1) and 10 [8] years, respectively. Participation was higher among males compared to females, among older adults as compared to those aged from 18 to 44 years, and patients diagnosed 5 years ago and above (Table 1). Finally, 450 participants (63% out of 719 with a sex ratio of 0.76) were included in the statistical analysis. Their characteristics are shown in Table 2.

**Table 1:** Distribution of responses to phone calls according to demographic and disease data

		Unreachable	Refusals	Participant	p (p value)
Sex; n = 691	Female (414)	32.4	6.0	61.6	0.030
	Male (277)	23.1	6.5	70.4	
Age (years); 676 Median = 56.7 IQR = 19.3	18–44 (154)	38.3	3.9	57.8	0.032
	45–54 (152)	25.7	4.6	69.7	
	55–64 (194)	22.2	7.7	70.1	
	65–90 (176)	27.3	6.8	65.9	
Disease duration (years); 671 Median = 2.8 IQR = 5.4	0.5–0.9 (214)	33.2	6.5	60.3	0.001
	1–1.9 (79)	34.2	2.5	63.3	
	2–4.9 (172)	30.8	6.4	62.8	
	5–47 (206)	16.0	5.8	78.2

**Table 2:** Participants’ characteristics

		n	*%*
Sex; *n* = 450	Female	255	56.7
	Male	195	43.3
Age (years); 447 Median = 56.7 IQR = 18,0	18–44	89	19.9
	45–54	106	23.7
	55–64	136	30.4
	65–90	116	26.0
Disease duration (years); 448 Median = 2.8 IQR = 7.0	0.5–0.9	129	28.8
	1–1.9	50	11.2
	2–4.9	108	24.1
	5–47	161	35.9
Insurance policy; 450	Non-Insured Does not know	202	44.9
	Knows an Insurance	105	23.3
	Insured Person	143	31.8
National Health Insurance Scheme process; 450	Does not know	341	75.8
	Knows	58	12.9
	Enlisted	51	11.3
Diabetes and cardiovascular disease will be covered; 445	No	16	3.6
	Yes	51	11.5
	Does not know	378	84.9
Education Level; 446	Not Educated	122	27.4
	Primary	100	22.4
	Secondary	162	36.3
	Superior	62	13.9
Occupation; 449	None or Housewife	161	35.9
	Informal Sector	105	23.4
	Private or Public Employee	96	21.4
	Retired	87	19.4

**Table 3:** Prediction of premium participants were willing to pay according to sex: multinomial logistic regression (other variables were not significant)

Group of amount (euros per month)	Variable = sex	n (%)	Coefficient	Standard error	p	Odds Ratio [CI 95%]
< 4.5 (reference)	Female	69 (*27.1*)				
	Male	31 (*15.9*)				
4.5 or 7.5	Female	77 (*30.2*)	−0.81	0.27	0.003	0.44 [0.26–0.75]
	Male	78 (*40.0*)	0			
	intercept		0.92	.21	0.000	
15	Female	80 (*31.4*)	−0.40	0.28	0.152	0.67 [0.39–1.16]
	Male	53 (*27.2*)	0			
	intercept		0.54	0.23	0.018	
30 or 45	Female	29 (*11.4*)	−0.93	0.33	0.005	0.40 [0.21–0.76]
	Male	33 (*16.9*)	0			
	intercept		0.06	0.25	0.803	

**Fig. 2:**
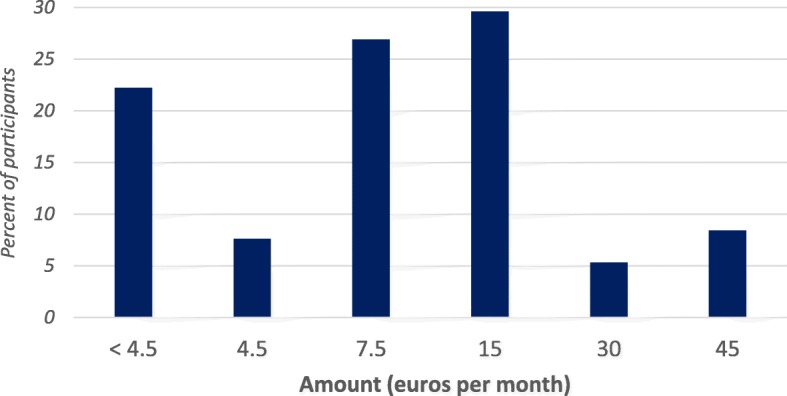
premium that 450 participants stated to be willing to pay

The frequency distribution of amount per month the participants stated they were willing to pay for an upcoming public health insurance scheme to ensure the sustainability of health care services they may need to face their condition is graphically presented in Fig. 2. Over one-fifth (22.2%) of participants were not willing to pay at least 4.5 euros per month while almost one third and three out of ten participants stated to be willing to pay 4.5 or 7.5 and 15 euros per month, respectively. In other words, 43.3% [38.8–48.0] of participants were willing to pay 15 euros or above per month. Among the 692 target patients alive at the time of their interview, this proportion was 28.2%. In an univariate analysis (data not shown), men were more likely to pay a higher amount as compared to women (*p* = 0.006), informal sector workers, private or public employees and retired were more likely to pay a higher amount as compared to jobless participants and housewives (*p* = 0.029).

Multinomial logistic regression returned only sex as a predictor of the amount per month participants would be willing to pay (Table 3). Overall, females were less willing to pay high amounts, though, the differences were significant for two modalities, 4.5 or 7.5 and 30 or 45 euros as compared to the reference modality below 4.5 euros. In fact, males were 2.3 and 2.5 times more likely to be willing to pay 4.5 or 7.5 and 30 or 45 euros, respectively. However, odds ratio corresponding to the modality 15 euros was less strong and not statistically significant suggesting that males were not more likely than females to state being willing to pay a premium of 15 euros for public health insurance scheme as compared to the participants in the reference modality below 4.5 euros.

## Discussion

Overall, a high percentage of the diabetic and cardiovascular patients participating in this study were willing to pay for an upcoming national health insurance scheme, assuming that substantial revenues could be mobilized to finance the goods and services currently provided by university teaching hospitals in Côte d’Ivoire. The premium amount the participants stated they could afford to pay is to a certain extent higher than those previously elicited in other studies in West Africa [5, 10, 11] and recent study in the Kingdom of Saudi Arabia, a high-income developing country with a very high Human Development Index (0.847 > 0.800) and the only Arab country to be part of the G20 (Group of Twenty) major economies [6]. We could not exclude starting point bias with the bidding game technique used in this study, but it is noticeable that even if the starting point was set high, over one-tenth of individuals stated they could afford to pay over the starting point in this sample with a moderate socioeconomic status. Similarly to this study, another study in Nigeria elicited that HIV-infected or AIDS patients were willing to pay the same levels of premium amount for antiretroviral drugs [12].

Understanding how the main influencing factors are associated with the willingness to pay would help to facilitate the establishment and implementation of a set of contribution people will be asked to achieve. In fact, it is expected that higher pooled funds will enable health financing manager to extend coverage to individuals and services that were not previously covered and reduce direct payments needed for each service [13].

The association of sex with the premium amount the participants stated to be willing to pay was found varying according to the premium amount. In fact, in the lower and higher amounts, female’s willingness to pay premium was lesser than the male’s one, but in the intermediate amount, there was no statistically significant difference. Sex was found to be associated with the willingness to pay for health insurance in a lot of previous studies carried out in West Africa [5, 10, 14]. It was exposed that there was no significant difference in willingness to pay between men and women if they could write or read, but there was a significant difference if they could not on one hand and unmarried women had higher willingness to pay than married on another hand [14]. In the context of this study, sex or gender influences other variables distribution such as education, occupation/income which were found predicting willingness to pay [10, 15, 16]. In addition, men’s and women’s willingness to pay are not affected by the same variables nor with the same way [14]. All these may result in a variation of the impact of sex on the willingness to pay pattern.

This study included patients who, mainly, were paying for healthcare they currently sought. These patients didn’t know whether diabetes and cardiovascular diseases will be covered by the upcoming national health insurance scheme which was, at the time of study, in a pilot phase with students. Thus the targeted insurance market didn’t exist and the implemented contingent valuation method could be used to monetize the extent to which study participants valued insurance along with their condition [17]. Moreover, due to a low number, some participants considered in higher socioeconomic status were excluded from analysis. Therefore, more substantial contribution could be expected since participating in the cited insurance scheme is mandatory. Furthermore, participants who stated not willing to pay the minimum premium proposed were added to those who stated to be unable to pay any contribution. In this way, we dealt firstly to avoid former premium information bias and secondly to highlight how extended was people’s acceptability to contribute to an upcoming health insurance scheme. Consequently, the participants’ maximum willingness to pay was not a continuous quantitative variable but indicated their classification. Then this dependent variable was analyzed with logistic regression. On another note, there were statistically significant differences in some individual characteristics (sex, age, and disease duration) between the participating persons and the non-participating persons. This last group was divided into unreachable and refusals. The younger and more recent sick were likely to be unreachable and the older were likely to refuse to participate. These non-participating persons may comprise a lot of kind of individuals such as indigent ones. These indigent non-participating patients with chronic diseases together with participants really unable to contribute need exemption mechanisms to be applied [18].

The willingness to pay studied here is useful for setting premiums when setting up a health insurance scheme, but it remains an element of health system strengthening that includes financial provisions for the health system to achieve the goal of universal health coverage, including protection against financial risks, access to quality health services and access to safe, effective, high-quality and affordable essential medicines and vaccines for all [19, 20]. In addition, it would be useful to provide information on changes in the welfare of beneficiaries through information on trade-offs between the attributes of a health insurance system, such as benefit packages, service providers, drug and test coverage, and willingness to pay premiums [21]. At present, the results of this study show that there is potential to support the future health insurance scheme through member contributions. An important issue from the perspective of policy makers is to set the range of premium amounts to be collected. Because of the participation and premium amount patterns described above, the economic relevance of the WTP estimates suggested from this study supports the viability of social health insurance in the country.

## Conclusions

This study suggests that it may be possible for the government to implement a contributory national health insurance scheme sustainable if it could engage in social marketing strategies and provide strategic information to raise awareness and enhance equitable maximum participation. Further research covering the whole population of the country to get more comprehensive results about “the rich subsidize the poor and the healthy subsidize the sick” would produce a broader pattern of willingness to pay and a large pool of premiums to reach the objective of financial risk protection.

## Additional files

Additional file 1: Accord_participation_revu <anonymous demographic form> (DOCX 59 kb)

Additional file 2: Questionnaire_DAP.qes < pre-tested interviewer-administered questionnaire> (QES 3 kb)

## Abbreviations

%: Percentage
CI 95%: Confidence interval 95%
FCFA: Franc Communauté financière d’Afrique
G20: Group of twenty
GDP: Gross domestic product
IQR: InterQuartile range
ln: Natural logarithm
MUGEF-CI: Mutuelle générale des fonctionnaires et agents de l’État de Côte d’Ivoire
n: Number
p: *P* value
P: Probability
WHO: World Health Organization
WTP: Willingness to pay

## Authors’ information

All authors hold firstly an academic position and second position to tertiary level health facilities
