# Supplementary material for: Diabetic and cardiovascular patients’ willingness to pay for upcoming national health insurance scheme in Côte d’Ivoire
Source: Health Econ Rev. 2019 Mar 8;9:8. doi: 10.1186/s13561-019-0225-y (PMC6734508; doi:10.1186/s13561-019-0225-y)
Supplement: Supplementary file 1 — Accord_participation_revu (DOCX 59 kb) [file 13561_2019_225_MOESM1_ESM.docx]

**ACCORD DE PARTICIPATION A UNE ETUDE**

Pour contribuer à la réussite de l’assurance maladie publique, vous êtes invité(e) à participer à une étude portant sur la cotisation que les personnes sont prêtes à payer pour bénéficier de la prise en charge des frais de santé en Côte d’Ivoire ; par interview téléphonique (recherche scientifique universitaire)

**PRISE DE RENDEZ-VOUS TELEPHONIQUE**

Numéro de téléphone: /___/___/___/___/___/___/___/___/

Jours de la semaine pour l’appel:

Heures d’appel préférées:

**DONNEES SUR LE PATIENT**

Sexe: femme |__| homme |__|

Date de naissance: __ __ / __ __ / __ __ __ __

Date, mois et/ou année de découverte de la maladie*****:

__ __/ __ __/ __ __ __ __ ******Diabète, Maladies Cardio-Vasculaires*

Site:

**NB** : Nous nous engageons à garantir la confidentialité de vos données et ne les utiliser que pour cette étude

**ACCORD DE PARTICIPATION A UNE ETUDE**

Pour contribuer à la réussite de l’assurance maladie publique, vous êtes invité(e) à participer à une étude portant sur la cotisation que les personnes sont prêtes à payer pour bénéficier de la prise en charge des frais de santé en Côte d’Ivoire ; par interview téléphonique (recherche scientifique universitaire)

**PRISE DE RENDEZ-VOUS TELEPHONIQUE**

Numéro de téléphone: /___/___/___/___/___/___/___/___/

Jours de la semaine pour l’appel:

Heures d’appel préférées:

**DONNEES SUR LE PATIENT**

Sexe: femme |__| homme |__|

Date de naissance: __ __ / __ __ / __ __ __ __

Date, mois et/ou année de découverte de la maladie*****:

__ __/ __ __/ __ __ __ __ ******Diabète, Maladies Cardio-Vasculaires*

Site:

**NB** : Nous nous engageons à garantir la confidentialité de vos données et ne les utiliser que pour cette étude
